# The impact of acquired coagulation factor XIII deficiency in traumatic bleeding and wound healing

**DOI:** 10.1186/s13054-022-03940-2

**Published:** 2022-03-24

**Authors:** Christian Kleber, Armin Sablotzki, Sebastian Casu, Martin Olivieri, Kai-Martin Thoms, Johannes Horter, Felix C. F. Schmitt, Ingvild Birschmann, Dietmar Fries, Marc Maegele, Herbert Schöchl, Michaela Wilhelmi

**Affiliations:** 1 Medical Center, Orthopaedic, Trauma and Plastic Surgery Clinic, University of Leipzig, Leipzig, Germany; 2 The Clinics of Anesthesiology, Critical Care and Pain Therapy, Klinikum St. Georg GmbH Leipzig, Delitzscher Strasse141, 04129 Leipzig, Germany; 3 Emergency Department, Asklepios Hospital Wandsbek, Hamburg, Germany; 4 Pediatric Thrombosis and Hemostasis Unit, Dr. Von Hauner Children’s Hospital, LMU Klinikum, Munich, Germany; 5 Department of Dermatology, University Medical Center Goettingen, Robert-Koch-Strasse 40, 37075 Goettingen, Germany; 6 BG Klinik Ludwigshafen, Ludwig-Guttmann-Str. 13, 67071 Ludwigshafen, Germany; 7 Department of Anaesthesiology, Heidelberg University Hospital, Heidelberg, Germany; 8 Heart and Diabetes Center of Nordrhein-Westfalen, Institute of Laboratory and Transfusion Medicine, University Hospital of the Ruhr-University of Bochum, Bad Oeynhausen, Germany; 9 Department of General and Surgical Intensive Care Medicine, Medical University Innsbruck, Innsbruck, Austria; 10 Department of Traumatology and Orthopedic Surgery, Cologne Merheim Medical Center (CMMC), Witten/Herdecke University, Campus Cologne-Merheim, Cologne, Germany; 11 Department of Anaesthesiology and Intensive Care Medicine, AUVA Trauma Centre Salzburg, Academic Teaching Hospital of the Paracelsus Medical University, Salzburg, Austria; 12 Ludwig Boltzmann Institute for Experimental and Clinical Traumatology, Vienna, Austria; 13 Trauma Department, Hannover Medical School, Hannover, Germany

**Keywords:** Factor XIII, Wound healing, Acquired bleeding, Surgery, Factor XIII deficiency

## Abstract

Factor XIII (FXIII) is a protein involved in blood clot stabilisation which also plays an important role in processes including trauma, wound healing, tissue repair, pregnancy, and even bone metabolism. Following surgery, low FXIII levels have been observed in patients with peri-operative blood loss and FXIII administration in those patients was associated with reduced blood transfusions. Furthermore, in patients with low FXIII levels, FXIII supplementation reduced the incidence of post-operative complications including disturbed wound healing. Increasing awareness of potentially low FXIII levels in specific patient populations could help identify patients with acquired FXIII deficiency; although opinions and protocols vary, a cut-off for FXIII activity of ~ 60–70% may be appropriate to diagnose acquired FXIII deficiency and guide supplementation. This narrative review discusses altered FXIII levels in trauma, surgery and wound healing, diagnostic approaches to detect FXIII deficiency and clinical guidance for the treatment of acquired FXIII deficiency.

**Supplementary Information:**

The online version contains supplementary material available at 10.1186/s13054-022-03940-2.

## Introduction

**Fig. 1:**
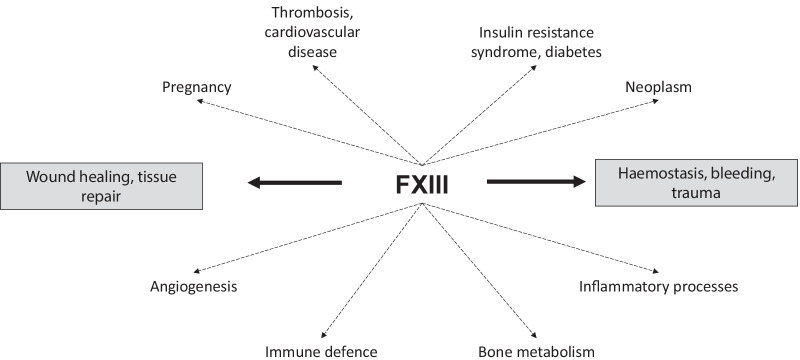
Overview of FXIII functions

**Fig. 2:**
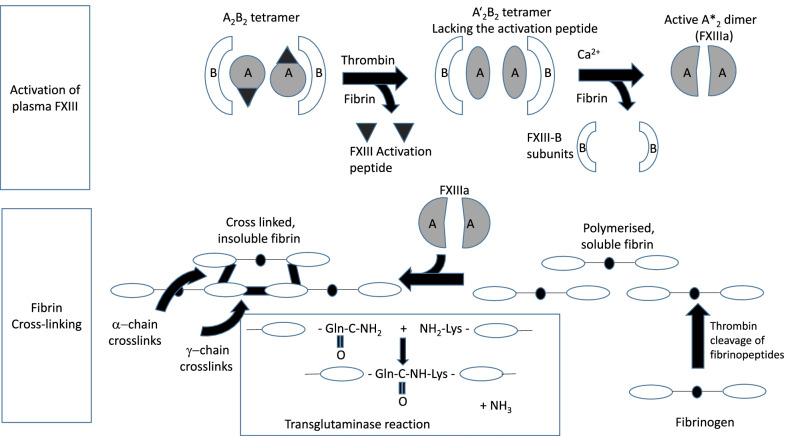
Activation and action of plasma FXIII (From [7], © 2012 International Society on Thrombosis and Haemostasis. Reprinted with permission). FXIII circulates in plasma consisting of two catalytic A subunits and two carrier B subunits. Thrombin (Factor IIa) performs the activation step of FXIII, where thrombin cleaves the activation peptide from the A subunits before Ca^2+^ ions cause dissociation of the subunits. The result is a dimer of two activated FXIIIa A subunits. Presence of fibrin/fibrinogen enhances both FXIII activation steps. Following activation, FXIIIa cross-links lysine (Lys) and glutamine (Gln) residues of fibrin α- and γ-chains leading to a three-dimensional network of insoluble fibrin molecules

Factor XIII (FXIII) is an enzyme of the coagulation cascade which plays a key role in maintaining the functional integrity of fibrin clots [1]. Additionally, FXIII has a range of other functions, including wound healing and tissue repair (Fig. 1). FXIII circulates in plasma as a protransglutaminase comprising two catalytic A and two carrier B subunits [1–3] and intracellularly as a homodimer of constitutively active A subunits [4]. Plasma FXIII is activated by thrombin which cleaves the activation peptide from the A subunits; binding of Ca^2+^ ions and fibrin substrate results in a conformational change exposing the active site [1–3]. After tissue injury and fibrin clot formation, thrombin-activated FXIII catalyses covalent cross-linking of fibrin chains, stabilising the clot [1]. FXIII also exerts antifibrinolytic activity through cross-linking of α_2_-antiplasmin to fibrin (Fig. 2) [5–7].

**Fig. 3:**
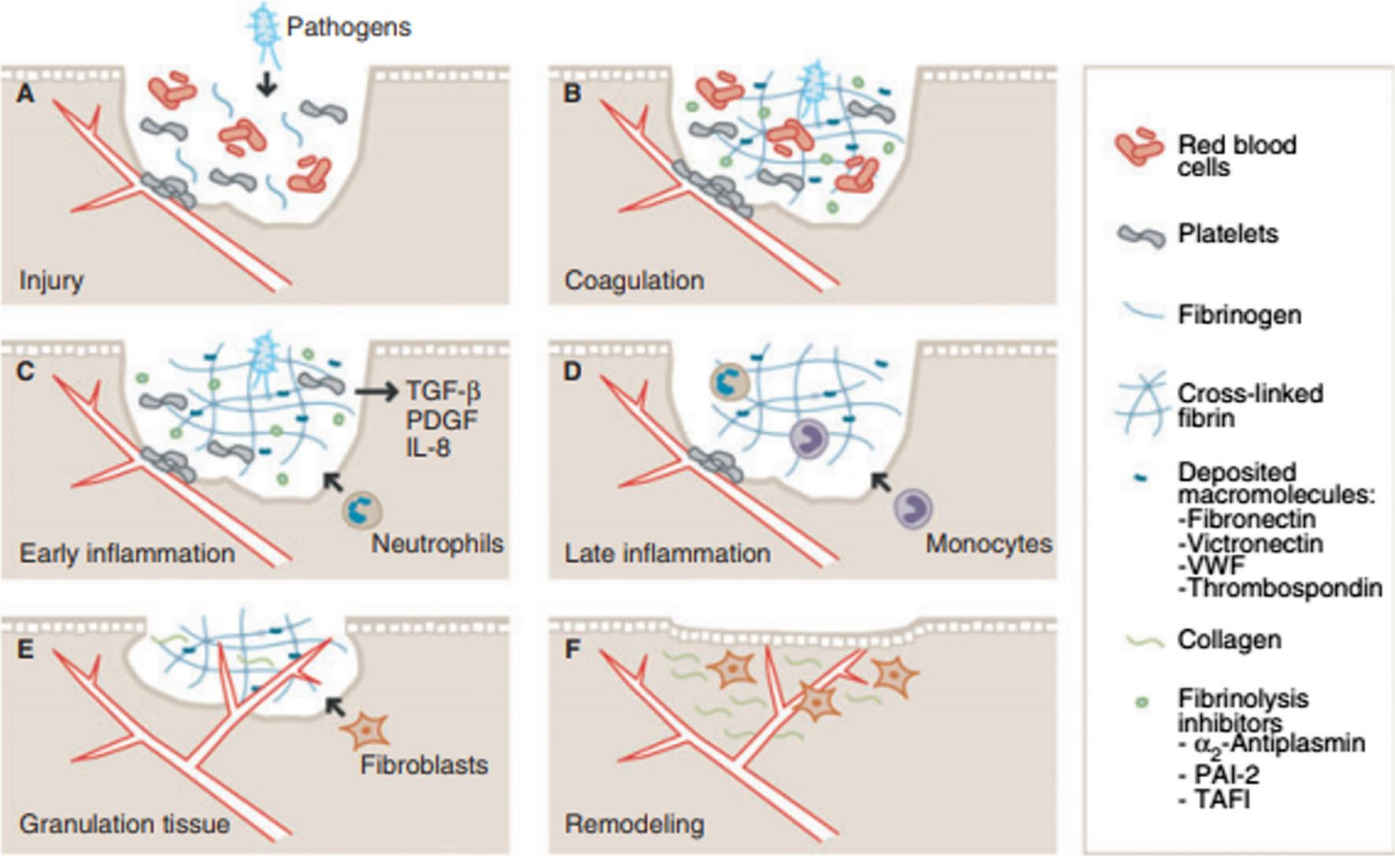
The roles of plasma FXIII (pFXIII) during wound healing. **a** Following injury and leakage of blood constituents into the wound, pFXIII enhances aggregation of platelets to the injured endothelium, limiting exudation. **b** Activated pFXIII (pFXIIIa) mediates cross-linking of the deposited fibrin, structural macromolecules, fibrinolytic inhibitors, and invading pathogens. **c**, **d** Inflammatory mediators secreted by, for example, platelets and epithelium lead to recruitment of neutrophils, monocytes and additional leukocytes into the wound. FXIIIa-induced cross-linking of the provisional matrix creates the basis for their integrin-mediated interactions with the provisional matrix. This enhances cellular invasion of the wound, and stimulates leukocyte survival and proliferation. **e** During granulation tissue formation, fibroblasts and endothelial cells (ECs) invade the wound, facilitating collagen deposition and angiogenesis. They are guided through integrin interactions with cross-linked macromolecules in the wound, and this stimulates their migration and survival. FXIIIa stimulates angiogenesis and granulation tissue formation by mediating complex formation of endothelial α_V_β_3_ and vascular endothelial growth factor receptor 2 on ECs. **f** Through the ongoing remodelling phase, the normal tissue architecture is re-established. IL, interleukin; PAI-2, plasminogen activator inhibitor-2; PDGF, platelet-derived growth factor; TAFI, thrombin-activatable fibrinolysis inhibitor; TGF-b, transforming growth factor-b; VWF, von Willebrand factor. From [9], ©2013 International Society on Thrombosis and Haemostasis. Reprinted with permission)

The role of FXIII in wound healing is known from preclinical studies (Fig. 3) [8, 9]. In FXIII-deficient mice, excisional wound healing was delayed compared to controls or FXIII-deficient mice receiving FXIII supplementation [10]. Moreover, FXIII cross-links fibrin to surface proteins of invading bacteria (e.g. Streptococcus [11], Staphylococcus and Escherichia [12]), trapping bacteria in the clot and reducing the risk of tissue infection [9, 11, 12]. FXIII also binds to α_v_β_3_ integrin, resulting in VEGFR-2 activation, promotion of endothelial cell proliferation and survival, and angiogenesis [13].

FXIII activity varies between individuals; activities as high as 250% of the reference range have rarely been reported [14]. Inherited FXIII deficiency is graded according to FXIII activity into (1) severe: undetectable FXIII associated with spontaneous major bleeding, (2) moderate: < 30% FXIII activity associated with mild spontaneous or triggered bleeding, and (3) mild: > 30% FXIII activity, usually asymptomatic. Plasma FXIII levels in acquired FXIII deficiency are typically 30–70%, but a clear association with spontaneous bleeding complications has not been demonstrated [15]. Lower-than-reference FXIII levels prior to, or hyper-consumption during and after, trauma or surgery, may be associated with increased bleeding risk [16]. Potential causes include immune-mediated inhibition and non-immune hyper-consumption or hypo-synthesis. Acquired FXIII deficiency may likewise be idiopathic or linked to co-morbidities such as malignancies or autoimmune diseases (Table 1) [17].

**Table 1:** Causes of acquired FXIII deficiency. Adapted from Yan et al. [17]

	Pathophysiology	Associated conditions
Immune	Autoantibody-mediated inhibition or rapid clearance	Autoimmune disease Systemic lupus erythematosus (SLE) Rheumatoid arthritis Malignancy Solid tumours Haematologic malignancy Medications Isoniazid Monoclonal gammopathy of undetermined significance (MGUS)
Non-immune	Hyper-consumption	Surgery Disseminated intravascular coagulation (DIC) Inflammatory bowel disease Henoch-Schoenlein purpura (HSP) Sepsis Thrombosis Pulmonary embolism, stroke
	Hypo-synthesis	Liver disease Leukaemia Medications Valproate Tocilizumab

This narrative review considers the clinical evidence for the role of FXIII in wound healing and trauma in a range of settings and provides the authors’ opinion on diagnosis and treatment of acquired FXIII deficiency. The diagnosis and treatment of congenital and immune-acquired FXIII deficiency, use of FXIII supplementation to correct defective cellular FXIII function, and use in paediatric patients, are beyond the scope of this review.

### Literature search

Due to limited availability of level 1 evidence and the variable nature of data reported, with differing outcome measures, a formal approach using PICO and GRADE methodology was not considered appropriate. Therefore, our aim was to summarise available clinical evidence. A literature search was conducted in PubMed on 21 January 2021. The search term factor XIII was combined with: trauma; complication; postpartum haemorrhage; cancer AND surgery; cardiac surgery; infection; sepsis; fistula; ulcer; burn; wound healing; diagnosis AND (test OR assay); (guidelines OR guidance OR recommendations OR algorithm). Exclusion criteria included: congenital FXIII deficiency; immune-mediated FXIII deficiency (with auto-antibodies); other clinical settings; fibrin glue; non-clinical studies; reviews; articles in a language other than English or German; full text not available.

Overall, 789 articles were retrieved, including 129 duplicates, leaving 660 articles. Following screening to identify relevant clinical studies with sufficient patient numbers and adequate reporting of results, 563 articles were excluded (Additional file 1: Fig. S1). The authors reviewed all 97 resulting articles to identify 18 papers (Table 2) [18–35].

**Table 2:** Summary of key papers involving clinical experience with FXIII

	References	Year	Indication	Study type	Patients	Protocol	Key outcomes
Trial 1	Baer et al. [18]	1980	Wound healing (surgery)	Randomised controlled trial	95 patients undergoing major abdominal or vascular surgery or patients with risk factors for development of wound healing disturbances	15 patients had FXIII levels within reference ranges whilst 80 patients had a pre- or post-operative deficit in FXIII (< 87.5%) and were randomised to receive either FXIII supplementation or no supplementation according to the formula: FXIII units for supplementation = 0.5 × body weight x FXIII deficit (%)	All patients showed a post-operative decrease in FXIII levels, the incidence of wound healing disturbance correlated with decreased FXIII levels, severity of FXIII decrease correlated with severity of surgical intervention and supplementation with FXIII concentrate reduced the incidence of post-operative surgical complications FXIII supplementation: 10 wound healing disorders/38 patients No supplementation: 19 wound healing disorders/42 patients
Trial 2	Chaoui et al. [19]	1999	Wound healing (surgery)	Randomised controlled trial	100 patients after surgery of head/neck tumours	1250 IU FXIII on post-operative day 2, 4 and 6 or no FXIII supplementation Results are displayed for patients with high-risk for wound healing disorders (17 controls, 20 FXIII supplementation)	FXIII plasma levels not predictive of wound healing disturbances. In FXIII group, plasma levels increased by ~ 30% (not significant) 10/20 (50%) of patients had primary wound healing compared to 7/17 (35%) controls
Trial 3	Fujita et al. [20]	2006	Wound healing (surgery)	Single-centre, open-label study	17 patients with anastomotic leaks (n = 16) or non-healing fistulas (n = 1) after gastrointestinal surgery who had serum protein and albumin levels within reference ranges but low FXIII activity after the resolution of post-operative acute inflammation	A 240 U dose of FXIII concentrate was administered intravenously for 5 days	Improvement following treatment was observed in 15 cases (88.2%). FXIII activity increased to > 70% of the reference range in 11 cases (64.7%) but was 40–70% of the reference range in six cases (35.3%). Levels of plasma EGF and TGF-β increased in patients with improvement but did not change in patients without improvement
Trial 4	Gerlach et al. [21]	2000	Wound healing (surgery)	Retrospective study	1264 patients underwent intracranial operations	FXIII testing was carried out post-operatively in 34 patients in whom coagulopathies were suspected despite platelets, fibrinogen, prothrombin and partial thromboplastin time being within the reference ranges	Of the 1264 patients, a total of 20 patients (1.6%) had a post-operative haemorrhage; of the 34 patients with suspected coagulopathies and FXIII post-operative testing, 11 suffered a major post-operative haemorrhage. Levels of FXIII were within the reference ranges (> 60%) in 26 of the 34 patients whilst FXIII deficiency (< 60%) was found in eight patients. All eight patients with FXIII deficiency had a major post-operative haemorrhage whilst of the remaining 26 patients with FXIII levels within the reference ranges only three had a post-operative haemorrhage (p < 0.00001)
Trial 5	Gierhake et al. [22]	1974	Wound healing (surgery)	Double-blind, placebo-controlled trial	300 patients undergoing abdominal surgery	Patients received one dose of FXIII or placebo, 1 day pre-operatively and on day 1 to 3 post-operatively; the first 200 patients received 500 IU FXIII or placebo at each time-point and the next 100 patients received 750 IU FXIII or placebo	7.8% of patients in the FXIII group and 18.5% of patients in the placebo group developed wound healing disturbances (statistically significant, p < 0.01), post-operative FXIII levels fell further in the placebo group (62.3%) compared to the FXIII group (88.7%)
Trial 6	Muto et al. [23]	1997	Wound healing (surgery)	Multi-centre, open, randomised controlled study	310 patients: 196 with non-healing post-operative wounds and 114 with fistulae	Patients had FXIII levels < 70%, each received 1500 IU FXIII concentrate over 5 days or no treatment (controls)	In non-healing post-operative wounds and fistulae, the rate of improvement was significantly higher for patients treated with FXIII compared to controls (non-healing wounds 72% vs 32%; p < 0.01; fistulae 69% vs 38%, p < 0.01)
Trial 7	Mishima et al. [24]	1984	Wound healing (surgery)	Controlled, randomised, open trial	71 patients with post-operative wound healing disturbance e.g. fistulae, dehiscence of skin wounds without improvement after at least 14 days and with FXIII plasma levels < 70% at 3–5 days prior to inclusion	5 days after inclusion subjects were randomised to three groups: Control: no factor XIII supplementation; Group 1: FXIII 750 IU/day on days 6, 7 and 8; Group 3: FXIII 1500 IU/day on days 6, 7 and 8	Improvements occurred when FXIII plasma levels were > 70% whilst subjects with < 50% FXIII deteriorated; rate of relevant general improvement of wounds in blind evaluation: 61.9% (750 IU FXIII, Group 1); 76.2% (1500 IU FXIII, Group 2); 10.5% (no FXIII, control)
Trial 8	Schramm et al. [25]	1982	Wound healing (surgery)	Double-blind, randomised, placebo-controlled study	80 patients with cancer surgery of stomach, colon and rectum of which 55 patients were evaluable	Patients were randomised to receive either 1250 IU of FXIII immediately post-operatively and on post-operative days 2 and 4 or corresponding placebo	Overall, in the placebo group, 48.1% of patients had abdominal wall dehiscence or rupture compared to 35.7% in the FXIII group, a difference which was not statistically significant
Trial 9	Takeda et al. [26]	2018	Wound healing (surgery)	Randomised controlled trial	50 patients with post-operative pancreatic fistula	Patients randomly assigned in a 1:1 ratio to receive FXIII concentrate the day after randomisation or a control group that received no FXIII concentrate within 2 weeks	There was no significant difference in the duration of drain placement between groups, early administration of exogenous FXIII does not facilitate the healing of post-operative pancreatic fistula
Trial 10	Herouy et al. [27]	2000	Wound healing (ulcers)	Randomised, double blind, age-matched study	30 patients with venous leg ulcers that had been resistant to therapy and had existed for a mean of 1.5 years: 15 patients underwent FXIII treatment and 15 patients received placebo	Beside compression bandage therapy, wound surfaces were treated once daily with FXIII or with placebo (isotonic sodium chloride solution). Ulcers were debrided and topical treatment started 24 h later	A significant decrease in the wound surface was observed after topical treatment with FXIII compared with placebo with total closure of venous leg ulcers achieved within a mean of 6 weeks. A significant reduction in fibrinolytic activity was also observed after 96 h of FXIII treatment whilst the placebo group showed the typical activity of venous leg ulcers with no significant decrease in fibrinolytic activity
Trial 11	Wozniak et al. [28]	2001	Wound healing (chronic leg ulcer)	Open study	54 patients with ulcers of the lower extremity	Initial regimen was 2 × 250 IU FXIII in the first week; however, this was reduced to 1 × 250 IU FXIII in the first week, followed by 250 IU every 2 days in the second week and 250 IU every 3 days from the third week up to 6 weeks	Patients with venous ulcers due to post-thrombotic syndrome admitted after infections, pain or unsuccessful treatment required FXIII treatment in hospital for 3–15 weeks and could then continue out-of-hospital treatment
Trial 12	Peschen et al. [29]	1998	Wound healing (chronic leg ulcer)	Placebo-controlled clinical study	24 patients were placed into 2 groups each made up of 12 patients with leg ulcers > 1000 mm^2^ or < 1000 mm^2^	In each group 8 of 12 patients were given additional topical treatment of FXIII twice daily for 10 days (treatment groups)	Results demonstrate that locally applied FXIII promotes wound healing of more acute, smaller venous leg ulcers (< 1000 mm^2^); it is suggested that FXIII is inactivated rapidly after topical application since immunohistochemical staining of FXIII showed no significant difference before and after therapy
Trial 13	Erlebach et al. [30]	1999	Wound healing (burns)	Treatment report	93 patients with burns between 41 and 89% of body surface area	Patients with 41–60% burn area (n = 87) received 1250–2500 IU FXIII on the day of surgery and on the first two post-operative days after each surgery; patients with 61–89% burn area (n = 6) received 1250–2500 IU FXIII on the day of surgery and 1250 IU FXIII daily until wound healing was complete	FXIII at admission 61–78%, at day of first surgery (approx. day 3): 17–37%; Perceived benefits of treatment with FXIII were reduced blood loss and a lesser need for transfusion in addition to accelerated epithelial regrowth especially of skin harvesting areas
Trial 14	Godje et al. [31]	2006	Surgery	Double-blind placebo-controlled clinical trial	75 patients, after coronary surgery with extracorporeal circulation	2500 U FXIII, 1250 U FXIII and a placebo given to 25 patients each	FXIII administration decreases post-operative blood loss and the level of blood transfusion after coronary surgery; however, administration is only advantageous if plasma levels are below the reference ranges
Trial 15	Karkouti et al. [32]	2013	Surgery	Double-blind placebo-controlled clinical trial	409 cardiac surgery patients at moderate risk for transfusion	17.5 IU/kg (n = 143), 35 IU/kg (n = 138) or placebo (n = 128) after cardiopulmonary bypass	Administration of FXIII levels after cardiac surgery restored levels in patients at moderate risk for transfusion to pre-operative levels; replenishment of FXIII levels had no effect on transfusion avoidance, transfusion requirements or need for re-operation
Trial 16	Korte et al. [33]	2009	Surgery	Double-blind placebo-controlled clinical trial	22 patients were evaluated after elective gastrointestinal cancer surgery	FXIII (30 U/kg) or placebo	In patients at increased risk for intra-operative bleeding, clot firmness was decreased by ~ 8% in patients that received FXIII and for patients in the placebo group it was reduced by 38% (p = 0.004); patients at high risk for intra-operative blood loss demonstrated reduced loss of clot firmness when FXIII was given early during surgery
Trial 17	Levy et al. [34]	2009	Surgery	Randomised controlled trial	35 patients were randomised to FXIII and 8 to placebo after cardiopulmonary bypass	11.9, 25, 35 or 50 IU/kg FXIII or placebo in a 4:1 ratio	In the FXIII group, dosing with 25–50 IU/kg restored FXIII levels to those seen pre-operatively with a tendency towards overshoot in the 50 IU/kg group and for post-operative FXIII replenishment 35 IU/kg may be the most appropriate dose
Trial 18	Gerlach et al. [48]	2002	Surgery	Prospective study	876 patients undergoing 910 neurosurgical procedures who developed 39 post-operative intracranial haematomas (4.3% of surgeries)	Prothrombin time, partial thromboplastin time, platelet count, fibrinogen, and FXIII activity were tested in each patient pre- and post-operatively	13/39 (33.3%) patients with post-operative haematoma had post-operative FXIII < 60% vs. 61/867 (7%) without (p < 0.01). The relative risk for a post-operative haematoma was increased 6.4-fold in patients with post-operative FXIII deficiency. The risk was increased 12-fold in patients who also had post-operative decreases in fibrinogen levels (< 1.5 g/L) and 9-fold in patients with platelet count < 150 × 10^9^/L and FXIII < 60%

### Clinical evidence

#### Trauma

Patients who have sustained severe trauma are at increased risk of trauma-induced coagulopathy (TIC), usually triggered by acute trauma-haemorrhagic shock (THS). THS leads to reduced blood volume, resulting in tissue hypoxia and risk of organ damage. Therefore, early identification and intervention is critical to prevent widespread organ damage [7, 31]. TIC is characterised by activation and consumption of clotting factors (notably fibrinogen), dilutional coagulopathy, hyperfibrinolysis and inflammation [36, 37]. In addition, significant loss and consumption of FXIII has been reported [36, 37]. Preclinical studies in models of THS and experimental burn have suggested that FXIII supplementation may ameliorate THS-induced organ failure [38, 39]. Furthermore, in preclinical studies using an experimental haemorrhagic shock model high doses of FXIII promoted effective haemostasis for trauma-associated coagulopathy in vitro and in vivo [40, 41]. FXIII also improved clot adhesion in wounds in a standardised bleeding model [41]. In vitro*,* FXIII improved clot strength and increased resistance to hyperfibrinolysis (measured by rotational thromboelastometry [ROTEM]) and significantly decreased bleeding and prolonged survival in vivo [42].

Although use of FXIII concentrate has been investigated clinically in major trauma patients, no double-blind, randomised trials could be identified. FFP contains approximately 1.2 U/mL of FXIII [43], Administration of FXIII concentrate (15 U/kg body weight) to major trauma patients with ongoing bleeding and FXIII activity < 60% as part of a goal-directed transfusion algorithm, led to a reduction in the proportion of patients requiring massive transfusions and an overall reduction of red blood cell (RBC) and fresh frozen plasma (FFP) transfusion [44]. However, with changes to acute trauma care over time, including novel goal-directed coagulation management strategies and modified surgical approaches, the specific contribution of FXIII to improved outcomes could not be confirmed [44]. FXIII administration was also part of coagulation factor (CF)-based algorithms guided by viscoelastic tests for reversal of TIC, where use of CF concentrates was superior in reducing organ failure, reversal of TIC and transfusion requirements compared to FFP only [45, 46]. In the RETIC trial [45], 27/50 patients (54%) in the CF group had plasma FXIII < 60% and either diffuse microvascular bleeding or massive bleeding requiring transfusion of > 3 U RBCs/hour and received FXIII concentrate (20 IU/kg) with every other fibrinogen dose. In the FFP group, 11 patients (25%) received FXIII as rescue therapy. In the Swiss observational study, application of a target haematocrit range was associated with increased use of FXIII (15/172 patients [9%] vs. 6/172 [3.5%], *p* = 0.004), with a reduction in the proportion of patients requiring RBC transfusions (11.6% vs. 29.7% of patients, *p* < 0.001) and the number of transfusions [46]. In both studies, FXIII was part of an algorithmic approach and it was not possible to identify the specific contribution of individual components.

Opinion and experiences differ regarding the timing and potential effects of FXIII deficiency in major trauma. One view is that FXIII deficiency may be a late (typically 2–3 weeks) complication of major trauma due to hepatic dysfunction; another is that FXIII deficiency occurs 3–7 days post-trauma, particularly in cases of major surgery. However, ~ 30% of trauma patients exhibit FXIII activity < 60% on admission with a clear association to the degree of blood loss suggesting FXIII activity decreases shortly after trauma [45].

Consequently, evaluation of FXIII activity should be conducted following trauma. Depending on clinical risk factors, magnitude of the traumatic impact and incidence of further bleeding, FXIII measurements may be required every 5–7 days or at shorter intervals in settings with higher dynamics and turnover, e.g. major trauma or severe burns. Conversely, lower risk patients may require less frequent monitoring as plasma FXIII has a half-life of 11–14 days [47].

#### Surgery

An association between lower FXIII levels and peri-operative blood loss has been reported [16, 48, 49]. FXIII deficiency is associated with post-operative haemorrhagic events and requirement for post-operative transfusion [21, 35, 50]. Observational data from neurosurgical patients identified a post-operative FXIII level < 60% as an independent risk factor for post-operative intracranial bleeding [21, 35]. In a retrospective study of 34 patients with suspected coagulopathy and recorded post-operative FXIII levels, eight patients had FXIII levels < 60% and all had a major post-operative haemorrhage compared to 3/26 patients with FXIII levels within reference ranges; this difference was highly significant [21]. In a prospective study post-operative haematoma occurred in 39/910 neurosurgical procedures. Thirteen patients (33.3%) with post-operative haematoma had post-operative FXIII levels < 60% compared with 61 (7%) without haematoma, a relative risk of 6.4 [35].

A retrospective study evaluated whether FXIII along with fibrinogen or platelet count affected the probability of intra-operative RBC transfusions in patients during non-cardiac surgery. In total, 443/1023 patients received RBCs; FXIII deficiency (FXIII level < 70%) was associated with a significantly increased probability of RBC transfusion, as was platelet count but not fibrinogen depletion [51]. An observational study of CFs after coronary artery bypass graft demonstrated that pre- and post-operative FXIII activity and plasma fibrinogen levels at two hours after surgery may inversely correlate with post-operative blood loss [50]. Furthermore, in 49 post-surgical patients with suspected acquired FXIII deficiency, FXIII levels < 50% were identified in 55% of patients. FXIII deficiency was associated with decreased haematocrit, increased transfusion requirement and delayed bleeding [52]. Conversely, treatment with FXIII reduced post-operative blood loss and blood transfusion after coronary surgery in patients where post-medication levels of FXIII ≥ 70% could be achieved [31]. FXIII administration was only beneficial if plasma levels were below reference ranges and it was suggested that plasma levels should be measured prior to FXIII administration [31].

A more recent prospective case control study evaluated potential risk factors for surgical re-exploration due to bleeding after elective cardiac surgery. Multivariate analysis revealed reduced FXIII activity was independently associated with surgical re-exploration [53]. Reduced post-operative FXIII activity was also significantly associated with increased 30-day mortality in univariate, but not multivariate, analysis [53]. Finally, in a prospective, randomised, proof-of-concept study in patients undergoing surgery for gastrointestinal cancer who were at risk of increased intra-operative blood loss (preoperative fibrin monomer > 3 µg/L), reductions in the secondary endpoints of blood loss and fibrinogen consumption were observed in patients who received FXIII [33].

Data from randomised controlled trials (RCTs) using recombinant FXIII (rFXIII) suggest that peri-operative FXIII supplementation has a varying impact on transfusion requirements; however, these trials were either unsuitable to evaluate FXIII or produced non-significant results [32–34]. In a study primarily designed to assess the safety and pharmacokinetics of rFXIII, there was less chest tube drainage in patients who received rFXIII following cardiopulmonary bypass (CPB) compared to placebo [34]. In a double-blind, placebo-controlled, multicentre trial involving 409 patients undergoing CPB surgery, rFXIII administration had no effect on blood transfusion requirement or re-operation compared to placebo; however, the cohort was limited to patients with a moderate risk for transfusion and transfusion avoidance was higher than anticipated with mean FXIII levels around 80% prior to drug administration. The efficacy of rFXIII may be better demonstrated in patients with a higher transfusion risk, in those with severe FXIII deficiency, or instead of antifibrinolytic drugs [32].

In post-operative intensive care patients with low FXIII and high fibrinogen levels, in vitro supplementation of FXIII to supraphysiological levels increased clot firmness and stability, and accelerated clot formation [42].

### Surgical wound healing

Prolonged reduction in plasma FXIII levels following surgery has been demonstrated [18, 23, 54, 55] and FXIII supplementation reduced the incidence of post-operative complications including disturbed wound healing [18, 23–25]. In 70% of patients with prolonged air leak following pulmonary lobectomy, air leaks resolved when FXIII was administered for five days. In patients who responded, post-operative FXIII levels were lower compared to those who did not respond to administration of FXIII indicating two potential mechanisms for prolonged air leak; one of which is related to FXIII consumption [54]. In an open-label, randomised study in 310 patients with disturbed post-operative wound healing or fistulae after surgery of the gastrointestinal tract, FXIII treatment for five days reduced wound size compared to controls [23]. Similarly, in an open-label study of 71 patients with post-operative wound healing disturbances, wound size at day 15 remained largely unchanged from baseline in controls, but decreased in the FXIII-treated groups; findings were similar for patients with fistulae [24]. In a double-blind placebo-controlled study of 55 patients undergoing laparotomy, 35.7% of patients in the FXIII group had wound healing complications compared to 48.1% of controls. This difference was not statistically significant, potentially due to the relatively small sample size [25].

### Non-surgical wounds

FXIII deficiency has also been associated with impaired healing of non-surgical wounds, such as leg ulcers [27–29, 56–58], burns [30, 59], and pressure sores [60]. Topical treatment of venous leg ulcers with FXIII has shown promising results in small trials and case studies [27–29, 56, 57]. In a study of 54 patients with ulcers of the lower extremities, including a subgroup with post-thrombotic ulcus cruris venosum that had pre-existed for a mean of 11.8 months, in-hospital treatment with FXIII for a mean of 3.15 weeks permitted out-of-hospital management, and skin transplantation could be accomplished in many cases [28]. Furthermore, locally applied FXIII promoted healing of acute, small leg ulcers (< 1000 mm^2^) in a trial of 24 patients [29]. Similarly, a randomised, double-blind, age-matched study in patients with venous leg ulcers confirmed a faster healing rate and significantly reduced lesional fibrinolytic activity with topical FXIII treatment compared to controls [27].

### Refractory wounds and fistulae

In line with the role of FXIII in limiting the spread of invading bacteria and promoting wound healing, FXIII levels were reduced in patients with post-operative non-healing fistulae or anastomotic leaks [20, 24, 61]. Furthermore, the use of FXIII concentrate has been shown to be beneficial for the treatment of post-operative wounds [20, 24, 61], as well as serious skin infections [62]. Thus, in a study evaluating FXIII treatment for post-operative, refractory wounds, improvements occurred in patients with FXIII levels > 70% while deterioration was observed in those with FXIII levels < 50% [24]. In a patient with decreased FXIII activity and multiple intractable enterocutaneous fistulae, FXIII administration after further surgery resulted in no fistula recurrence or other complications [61]. In another study, FXIII administration increased levels to > 70% in 64.7% of patients with anastomotic leaks or fistulae and low plasma FXIII activity levels; improved wound healing was associated with increased levels of plasma epidermal growth factor and transforming growth factor-β, and it was suggested that FXIII may accelerate wound healing through increasing circulating levels of growth factors [20].

Conversely, other studies have not demonstrated benefits from FXIII administration. In a single-centre study of 43 patients undergoing pancreatoduodenectomy, post-operative morbidity, including pancreatic fistulae, were comparable between patients with < 70% or ≥ 70% FXIII activity [48]. Similarly, in an RCT in 50 patients with post-operative pancreatic fistulae and FXIII levels ≤ 70%, early administration of FXIII concentrate did not facilitate fistulae healing [26].

### Burns

**Fig. 4:**
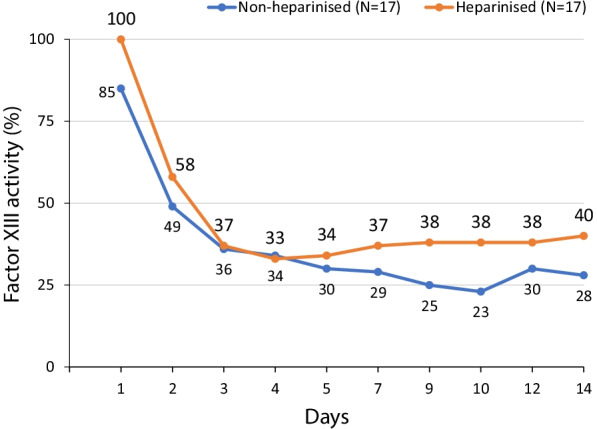
Factor XIII activity in non-heparinised and heparinised patients with severe burns. From [59] (©1977 Der Chirurg, adapted with permission). FXIII activity was measured daily from admission to general ward transfer in patients with severe burns (20–64% of body surface, N = 34). A rapid fall in FXIII activity was observed in the first three days in both heparinised and non-heparinised patients, where activity remained below 50% during 14-day follow-up for all patients

In 34 patients with severe burns (20–64% total body surface), there was a rapid fall of FXIII activity to ~ 35% during the first three days and this level remained < 50% until day 14 [59] (Fig. 4), suggesting that bleeding episodes, disturbed re-epithelialisation of mesh-graft harvesting areas and other clinical problems may be likely under these circumstances. Two studies have reported reduced transfusion requirements with FXIII supplementation in burn patients [30, 63]; the earlier study also reported reduced blood loss and accelerated epithelial growth, particularly around skin harvesting areas [30].

### Diagnosis

Abnormal bleeding may imply FXIII deficiency, but diagnosis requires specific laboratory tests [64]. Importantly, in cases of FXIII deficiency, routine coagulation tests such as prothrombin time, activated partial thromboplastin time, and thrombin time remain within their reference ranges, so it is essential to apply specific tests to assess FXIII levels [64, 65].

In 2011, the International Society on Thrombosis and Haemostasis (ISTH) recommended an algorithm for diagnostic testing to classify and grade congenital and acquired FXIII deficiencies [64]. In clinical practice standard activity tests based on transglutaminase activity (ammonia release and amine incorporation assays), isopeptidase activity or direct quantitative antigen assays are most commonly available [65]. Clot solubility tests may also be used, despite a lack of standardisation and inability to measure less prominent FXIII deficiency. Autoantibody assays may be performed if FXIII activity is below the reference range [65–67].

Clot strength and resistance to hyperfibrinolysis in vitro can be measured by ROTEM [36, 42] but direct measurement of FXIII by ROTEM has not, to-date, been possible [68, 69]. A study in cardiac surgery patients reported a close correlation between fibrinogen levels and FXIII and further suggested FXIII levels could be estimated using MCF-FIBTEM measured by ROTEM [70]. In addition, an unpublished study combined in vitro observation and FXIII measurement by ROTEM (EXTEM and INTEM) with a prospective evaluation in patients with severe trauma (personal communication C. Kleber). This approach requires confirmation and has not yet reached widespread clinical application.

### Clinical guidelines

Clinical guidelines for the management of acquired FXIII deficiency are limited. However, recommendations for the use of FXIII concentrate are covered in guidelines for the management of bleeding during surgery [71–73], postpartum haemorrhage [74], and following trauma [75]. Blood conservation guidelines from the US Society of Thoracic Surgeons state that FXIII concentrate may be considered after CPB in bleeding patients when other routine blood conservation measures prove unsatisfactory [71]. The European Society of Anaesthesiology recommends administration of FXIII concentrate (30 IU/kg) in cases of peri-operative bleeding and FXIII activity < 30% [72]. In contrast, the ISTH recommends against the use of FXIII concentrate for management of peri-operative bleeding, on the basis that the benefits remain unproven in the context of acquired FXIII deficiency [73]. The European guideline for the management of major bleeding following trauma suggests including FXIII monitoring in coagulation support algorithms and supplementation in bleeding patients with a functional FXIII deficiency [75]. However, there is no consensus on what level of FXIII indicates FXIII deficiency or when FXIII concentrate should be prescribed in these settings.

## Discussion

The limited inclusion of FXIII therapy into critical care guidelines suggests low awareness of potential FXIII deficiency in acquired bleeding and wound healing. Although data from RCTs remain limited, a considerable body of clinical evidence and expert knowledge has been accumulated over recent years on the use of FXIII in acquired bleeding and wound healing that could guide current treatment approaches. Due to the limitations of the published literature, we were unable to apply full systematic methodologies to evaluate the literature. However, every effort was made to accurately represent the data and the limitations therein. A supplementary search of clinicaltrials.gov and EU Clinical Trials register (conducted November 2021) found no ongoing clinical trials or substantive studies not already identified by our literature review.

It is the authors’ opinion that diagnostic measures concerning FXIII levels in the peri-operative setting are underrepresented and that there is less FXIII supplementation than current evidence would support. Increasing awareness of FXIII testing could help identify patients with acquired FXIII deficiency. Current recommendations for the management of FXIII deficiency are largely based on data from patients with congenital FXIII deficiency, which may not be transferable to patients with acquired FXIII deficiency in bleeding and wound healing. It is also not clear whether differences in the pathophysiological response to acute soft tissue trauma compared to elective surgery could affect the response to FXIII therapy. Therefore, tests should always be interpreted in the context of the clinical course of the disorder, concomitant diseases, operative/surgical trauma and bleeding characteristics and phenotype. In cases of ongoing bleeding with global test results and platelet counts within reference ranges, FXIII deficiency should be considered and a quantitative functional FXIII activity test performed. Although opinions and protocols may vary, a cut-off for FXIII activity of < 50–70% may be appropriate to diagnose acquired FXIII deficiency.

Current international treatment guidelines offer limited and seemingly contradictory recommendations regarding the use of FXIII in acquired bleeding and intensive care and some focus only on its use when congenital deficiency is present. Prices of FXIII products vary across Europe according to the source (plasma derived/recombinant) and licensed indications. However, costs are similar to other coagulation factor concentrates. Despite lack of consensus in clinical guidelines, the studies summarised in this narrative review indicate it may be beneficial to administer FXIII to patients with low FXIII levels. In the setting of active bleeding, prolonged wound healing, substantial soft tissue injury, or persistent infection/bacteraemia treatment with FXIII should be considered only if reduced activity is detected.

Regarding safety, FXIII is widely used to treat FXIII deficiency with few adverse events reported. The most common adverse events reported during use of Factor XIII Concentrate (Fibrogammin/Corifact; CSL Behring GmbH, Marburg, Germany) are allergoid-anaphylactoid reactions (e.g. generalised urticaria, rash, fall in blood pressure, dyspnoea) and rise in temperature, reported at an incidence of between 1/10,000 and 1/1000 [76]. The safety of Factor XIII Concentrate has also been confirmed through > 20 years of pharmacovigilance data, with a low risk of thromboembolic events reported over this period [77]. Although formal controlled trials in trauma are limited, studies in patients with acquired FXIII deficiency due to traumatic injury or post-operatively have indicated a good safety profile [21, 23, 24, 26, 28, 31, 32, 34]. Further information on the safety of FXIII in patients with severe trauma would be useful, although challenging to obtain due to the devastating nature of trauma and the multi-modal treatment employed.

### Future directions

FXIII is a haemostatic protein that stabilises blood clots and has a range of functions in wound healing, tissue repair, haemostasis, bleeding and trauma. A recent review has described three pools of FXIII within the circulation, in plasma, and within the cellular compartments of platelets and monocytes/macrophages, providing additional insights into the complex roles of FXIII in these physiological processes [78]. Although beyond the scope of this review, studies have indicated that FXIII levels/activity are impaired before and during extra-corporeal membrane oxygenation [79–81]. Moreover, acquired coagulopathy and reduced FXIII levels have been reported in patients with COVID-19 [82–84].

There is also increasing evidence that FXIII may be of use in areas beyond the scope of this narrative review, e.g. bone healing. Preclinical experimental models have demonstrated a positive effect during early stages of bone healing, with increased FXIII levels correlating with an increase in tensile strength and number of newly formed osteons [85, 86]. Studies in patients with inflammatory bowel disease suggest FXIII activity may vary according to disease activity [87–91] with initial clinical data suggesting a potential role for FXIII therapy – although results are conflicting [92–97].

There remain substantial research gaps and opportunities to further explore the role and use of FXIII in acquired bleeding and wound healing. Topical treatment as well as systemic application of FXIII in the impaired healing of non-surgical wounds merits further research and an RCT in this field would be of great interest. Further clinical studies are also vital in major abdominal surgery, septic infection and burn care to clarify the function of FXIII. Additionally, case series to identify risk factors for acquired FXIII deficiency would be of value, as would a prospective, multi-centre study with FXIII substitution dependent on activity cut-off, in patients that have bacteraemia and in trauma patients to leverage the knowledge on anti-infective properties. If such trials were conducted, a formal meta-analytical approach would be useful to confirm the utility of FXIII in specific clinical settings.

## Conclusions

In patients with low plasma FXIII levels, FXIII supplementation reduced the incidence of post-operative complications including delayed haemorrhage and disturbed wound healing. Several studies with positive outcomes have used ~ 60–70% activity as a trigger for FXIII supplementation. Increasing the awareness of FXIII testing could help identify patients with acquired FXIII deficiency in cases of acquired bleeding and delayed wound healing. Creating in-house standard operating procedures for the diagnosis and treatment of acquired FXIII deficiency could be highly beneficial.

## Supplementary Information

**Additional file 1: Fig. S1**. Literature search summary.

## Data Availability

Data sharing is not applicable to this article as no datasets were generated or analysed during the current study.

## Abbreviations

CF: Coagulation factor
CPB: Cardiopulmonary bypass
FFP: Fresh frozen plasma
FXIII: Factor XIII
rFXIII: Recombinant FXIII
RBC: Red blood cell
RCT: Randomised controlled trial
ROTEM: Rotational thromboelastometry
THS: Trauma-haemorrhagic shock
TIC: Trauma-induced coagulopathy
