# Supplementary material for: The impact of acquired coagulation factor XIII deficiency in traumatic bleeding and wound healing
Source: Crit Care. 2022 Mar 24;26:69. doi: 10.1186/s13054-022-03940-2 (PMC8943792; doi:10.1186/s13054-022-03940-2)
Supplement: Supplementary file 1 — Additional file 1: Fig. S1. Literature search summary. [file 13054_2022_3940_MOESM1_ESM.pptx]

## Slide 1
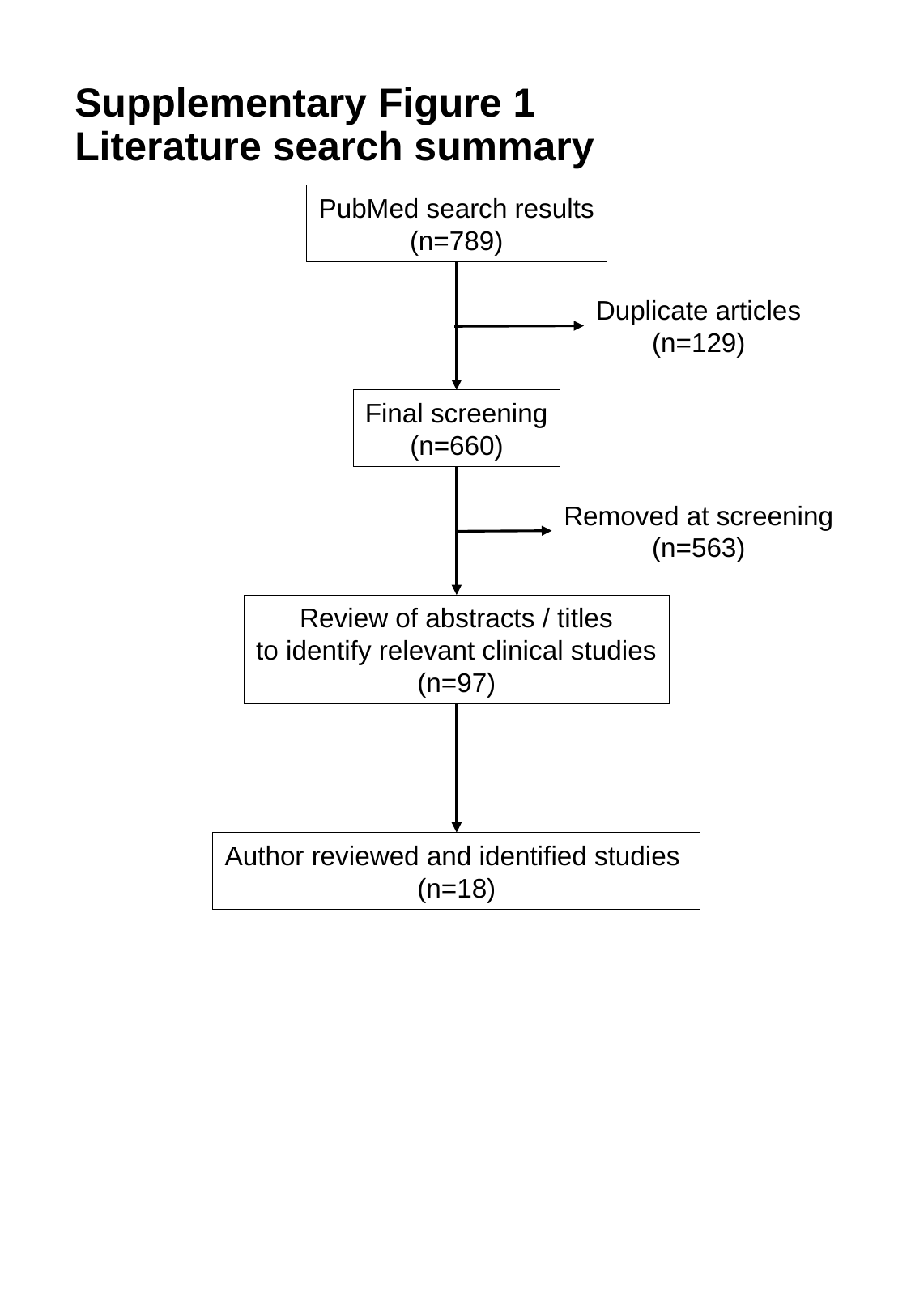

# Supplementary Figure 1 Literature search summary
PubMed search results
(n=789)
Duplicate articles
(n=129)
Final screening
(n=660)
Removed at screening
(n=563)
Review of abstracts / titles
to identify relevant clinical studies
(n=97)
Author reviewed and identified studies
(n=18)
